# The Use of Natural Polysaccharides as Biomaterials

**DOI:** 10.1155/2015/242378

**Published:** 2015-05-25

**Authors:** Paola Laurienzo, João C. Fernandes, Sylvia Colliec-Jouault, J. Helen Fitton

**Affiliations:** ^1^Institute for Polymers, Composites and Biomaterials, CNR, Via Campi Flegrei 34, 80078 Pozzuoli, Naples, Italy; ^2^Institute for Biomedical Imaging and Life Sciences (IBILI), Faculty of Medicine, University of Coimbra, 3000-548 Coimbra, Portugal; ^3^French Research Institute for Exploitation of the Sea (IFREMER), Microbial Ecosystems and Marine Molecules for the Biotechnology, BP 21105, 44311 Nantes Cedex 3, France; ^4^Marinova Pty Ltd., 249 Kennedy Drive, Cambridge, TAS 7170, Australia

The interest towards polysaccharides of natural origin is continuously growing during the past decade. Fields of interest for their applications are widening, ranging between ecocommodities, food supplements, cosmetics, pharmaceuticals, and biomedical uses. Exploitation of new sources of polysaccharides of different origin is well documented in recent literature. Since this tendency is involving biomaterials science in a pressing way, this journal set up to publish a special issue devoted to this topic. The result is a collection of twelve original research articles, whose authors belong to academic or research institutions of eleven different countries from Asia, Europe, and Australia. Papers are representative of a large share of biomedical applications, related chemical modifications, and manufacturing methodologies.

From a chemical point of view, the perspective is to replace traditional methods for production and modification of natural polysaccharides with more ecofriendly, efficient, and targeted methodologies. N. Chopin et al. from France (“A Direct Sulfation Process of a Marine Polysaccharide in Ionic Liquid”) described a new sulfation method to produce high specificity derivatives with biological activity. On the other hand, S. Islam et al. from Australia (“Comparison and Characterisation of Regenerated Chitosan from 1-Butyl-3-methylimidazolium Chloride and Chitosan from Crab Shells”) had exploited the effect of ionic liquid solvents on chemical-physical and functional properties of chitosan and demonstrated that ionic liquid solvents represent a good medium for dissolution of chitosan, also useful for blending with other polysaccharides.

J. Varshosaz et al. from Iran (“Preparation, Optimization, and Screening of the Effect of Processing Variables on Agar Nanospheres Loaded with Bupropion HCl by a D-Optimal Design”), A. Rees et al. from UK (“3D Bioprinting of Carboxymethylated-Periodate Oxidized Nanocellulose Constructs for Wound Dressing Applications”), and F. Hong et al. from China (“Preliminary Study on Biosynthesis of Bacterial Nanocellulose Tubes in a Novel Double-Silicone-Tube Bioreactor for Potential Vascular Prosthesis”) had focused on nanotechnology applied to polysaccharides, particularly on the use of sophisticated techniques to optimize the design of final products. J. Varshosaz et al. prepared agar nanospheres for controlled drug release using sophisticated Design-Expert software with the aim of optimizing the release characteristics by a careful control of fabrication conditions. A. Rees et al. reported on three-dimensional (3D) bioplotter applied to nanocellulose. 3D bioplotting allows the construction of complex shapes, otherwise unfeasible through traditional manufacturing techniques. The authors prepared nanocellulose in form of short nanofibrils of reduced viscosity and used this novel material as a bioink for printing 3D porous structures for wound healing applications. F. Hong et al. described a novel double tubes bioreactor, based on previous patents and papers of KLEMM group and GATENHOLM group, designed for the production of bacterial nanocellulose tubes for small vascular implants. Y. Shirosaki et al. from Japan (“Preparation of Porous Chitosan-Siloxane Hybrids Coated with Hydroxyapatite Particles”) developed a method for apatite deposition on a novel porous material based on chitosan, potentially interesting as bone substitute in craniofacial surgery. S. Uthaman et al. from Korea (“Polysaccharide-Coated Magnetic Nanoparticles for Imaging and Gene Therapy”) reviewed recent progresses of polysaccharide-coated magnetic nanoparticles for imaging and gene delivery, highlighting the role of polysaccharide coating and its advantages.

P. Montanucci et al. from Italy (“Insights in Behavior of Variably Formulated Alginate-Based Microcapsules for Cell Transplantation”) and S. Benni et al. from France (“Dynamic Contact Angle Analysis of Protein Adsorption on Polysaccharide Multilayer's Films for Biomaterial Reendothelialization”**)** explored the field of tissue engineering by investigating, respectively, the role of fabrication parameters on efficiency of alginate microcapsules for cell immobilization and the surface modification of stents by polysaccharides deposition to favor protein absorption and reendothelialization after implantation in human vessels. L. Russo et al. from Italy (“Hyaluronic Acid Based Hydrogels for Regenerative Medicine Applications”) developed novel hyaluronic acid hydrogels with mechanical properties similar to those of the ECM of natural tissues.

Polysaccharides have also been exploited for their potential healing effects. M. Matoba et al. from Japan (“Prevention of Polyglycolic Acid-Induced Peritoneal Adhesions Using Alginate in a Rat Model”) highlighted the antiadhesive effect of alginate in postoperative treatments to prevent adhesions induced by PGA meshes. P. K. Bhateja and R. Singh from India (“Antidiabetic Activity of* Acacia tortilis* (Forsk.) Hayne ssp. raddiana Polysaccharide on Streptozotocin-Nicotinamide Induced Diabetic Rats”) investigated the antidiabetic activity of a polysaccharide extracted from* Acacia tortilis*, a tree widespread in the globe, mainly in North Africa and Asia.

Uniformly, the authors highlighted the potentiality of this emerging class of biocompatible macromolecules in biomaterials field as source of both new molecules with therapeutic effects and new materials for regenerative medicine and realization of biomedical devices.

*Paola***  ***Laurienzo* *João***  ***C.***  ***Fernandes* *Sylvia***  ***Colliec-Jouault* *J. Helen Fitton*

